# Editorial: Integration of oral health care within the healthcare system

**DOI:** 10.3389/froh.2024.1387141

**Published:** 2024-03-18

**Authors:** Sourabha K. Patro

**Affiliations:** Department of Otolaryngology and Head and Neck Surgery, Postgraduate Institute of Medical Education and Research (PGIMER), Chandigarh, India

**Keywords:** oral health, integration of oral health, primary health care, WHO goals in oral health, community health, oral health for people

**Editorial on the Research Topic** Integration of oral health care within the healthcare system

It is really unfortunate that traditionally Oral Health has not been an integral part of primary health care. However, it is also an appreciable fact that in the last two–three decades the world has slowly started to realize the need to integrate oral health into the primary health care system. This can be evidenced by the *7th WHO Global Conference on Health Promotion - towards integration of oral health* in 2009, where the WHO largely supported the concept of integrating oral health into the health care system and mentioned five key tracks for the implementation of the same, namely: community empowerment; health literacy and health behavior; strengthening health systems; partnership and inter-sectoral action; and building capacity for health promotion (1). Population access to oral health care as a part of the health care system is limited by a number of challenges and concerns at three levels such as the micro level related to the individual seeking health care, the meso level related to the social processes and community structures at the local level, and the macro level related to the population-wide policies and establishments of a country, which need to be addressed for a smooth and fruitful integration of oral health care into the health care system (2).

Although it is completely logical to understand that health care needs in all forms, be they physical, mental, or spiritual, to be part of the health care system and that there should be policies to address this gap that exists traditionally, there have been attempts in the world to justify the need for incorporating and integrating oral health into the health care system as can be seen from the concepts of the common risk factor approach and the bidirectional relationship approach. The common risk factor approach does emphasize the evidence that the same risk factors (such as alcohol, smoking, poor hygiene, and injuries) are responsible for both non-communicable diseases and diseases related to oral health (3). The bidirectional disease approach emphasizes that oral health concerns and various other diseases can predispose to each other such as between diabetes and periodontal disease and between oral hygiene and associated concerns with oral lesions, cancer, etc. (4).

There have been various ways or approaches that have been proposed by various authors to describe the process of integrating oral health into the delivery of the health care system. Care coordination for case-based management at the primary care level by the primary care physician to ensure screening and referral as needed has been advocated for the last two decades, especially for children, and has been found to be effective (5, 6). The WHO as a part of its global goals for oral health 2020 emphasized building public awareness of knowledge and practices related to oral health promotion through various community programs at PHC (primary health care) levels and established the concept of stewardship in health care right at the primary care levels to ensure oral health care integration by hygienists (7–9). Also, for ensuring rightful education and training of caregivers in primary oral health care, there have been many proposals for including primary care rotations in the medical/dental school and residency training programs (10, 11). The integration of a diverse workforce mix at the primary care level, highlighting the importance of knowledge sharing, positions the Community Dental Health Coordinator as a pivotal orchestrator of oral health care (10).

Attempts have also been made to integrate oral health care practice and prevention through public-private partnerships and international collaborations such as the “Live Learn Laugh” program, which encourages public behavior to brush twice a day and use fluoride toothpaste (12, 13). In the process of collaborative approaches collaborations between medical and dental caregivers have been proposed to integrate oral health care in various ways such as co-location of medical care providers and dental hygienists, integration of dental hygienists into medical care teams, telehealth support at the primary care level with the help of communication technologies, and arrangements for basic oral health prevention and care services at the medical care facility with coordinated referral for better patient management (14). Collaboration goes a step further when care centers are coordinated through the collaboration of multiple specialties or health providers who learn from each other through interprofessional education and work closely with the patients and their families such as the collaboration of dentists, maxillofacial surgeons, otolaryngologists and craniofacial surgeons for complex and referred oral health care (15). Such settings can implement specialized care programs such as closed-loop referrals, interprofessional care programs (such as the “Into the mouths of babes” program in which pediatricians provide oral health care), and programs in which physicians and dental hygienists can be co-located. Another wonderful example of integrating oral health into the primary health care system is through the concept of “Accountable care organizations” where the health care system ensures that oral health preventive care providers are part of the primary health care team and that patient-centered care is provided (16).

Basic package oral care (BPOC) is a different approach that stratifies care into three categories: emergency treatment and referral, fluoride toothpaste and preventive services, and atraumatic restorative treatment using high-viscosity glass ionomers. This has been found to be effective in low-resource settings (17).

Despite the various ways of integrating oral health into the health care system that have been proposed the most considered approach to date is the Integration Framework Model. Integration is done at various levels and specialties in the form of public oral health programs, adaptive regulations, insurance support, and policy initiatives at the macro level; organizational integration such as co-location and interdisciplinary networks and professional integration in the form of interprofessional training, education, collaboration, etc. at the meso level; and patient-centered horizontal and vertical integration of caregiving at the micro level (18). However, available resources dictate the policies, hence there are various barriers in the process (19, 20), and thus, we are far from the ideal.

We explore the various new possibilities in this regard in this Research Topic.

## References

[1] Petersen P Kwan SJCDH. The 7th WHO Global Conference on Health Promotion-Towards Integration of Oral Health; 2009; Nairobi, Kenya (2010) 1(27). p. 129–36

[2] Harris R Raison H Christian B Bakare L Okwundu CI Burnside G. Interventions for improving adults’ use of primary oral health care services. Cochrane Database Syst Rev. (2017) 2017(8).

[3] Sheiham A Watt RG. The common risk factor approach: a rational basis for promoting oral health. Community Dent Oral Epidemiol. (2000) 28(6):399–406. 10.1034/j.1600-0528.2000.028006399.x 11106011

[4] Kapila YL. Oral health’s inextricable connection to systemic health: special populations bring to bear multimodal relationships and factors connecting periodontal disease to systemic diseases and conditions. Periodontol 2000. (2021) 87(1):11–6. 10.1111/prd.12398 34463994 PMC8457130

[5] Crall JJ. Development and integration of oral health services for preschool-age children. Pediatr Dent. (2005) 27(4):323–30. 16317973

[6] Betts KJ Moravec L. Integration of oral health screening, intervention, and referral into the pediatric well-child visit. J Pediatr Health Care. (2023) 37(6):609–15. 10.1016/j.pedhc.2023.05.007 37330729

[7] Monajem S. Integration of oral health into primary health care: the role of dental hygienists and the WHO stewardship. Int J Dent Hyg. (2006) 4(1):47–51. 10.1111/j.1601-5037.2006.00159.x 16451440

[8] Monajem S. The WHO’s action plan for oral health. Int J Dent Hyg. (2009) 7(1):71–3. 10.1111/j.1601-5037.2008.00325.x 19215314

[9] Petersen PE. Strengthening of oral health systems: oral health through primary health care. Med Princ Pract. (2014) 23 Suppl 1(Suppl 1):3–9. 10.1159/000356937 24525450 PMC5586948

[10] Mumghamba EG. Integrating a primary oral health care approach in the dental curriculum: a Tanzanian experience. Med Princ Pract. (2014) 23 Suppl 1(Suppl 1):69–77. 10.1159/000355520 24246734 PMC5586945

[11] Cully JL Thikkurissy S Sahay RD Harper R Lehmann C. Integration of a pilot oral health curriculum into a medical school pediatric rotation. Pediatr Dent. (2021) 43(4):258–61. 34467839

[12] Cohen LK. Live.learn.laugh.: a unique global public-private partnership to improve oral health. Int Dent J. (2011) 61 Suppl 2(Suppl 2):1. 10.1111/j.1875-595X.2011.00039.x 21770934 PMC9374986

[13] Bourgeois DM Phantumvanit P Llodra JC Horn V Carlile M Eisele JL. Rationale for the prevention of oral diseases in primary health care: an international collaborative study in oral health education. Int Dent J. (2014) 64 Suppl 2(Suppl 2):1–11. 10.1111/idj.12126 25209645 PMC9376526

[14] Braun PA Cusick A. Collaboration between medical providers and dental hygienists in pediatric health care. J Evid Based Dent Pract. (2016) 16 Suppl:59–67. 10.1016/j.jebdp.2016.01.017 27236997

[15] Atchison KA Weintraub JA. Integrating oral health and primary care in the changing health care landscape. N C Med J. (2017) 78(6):406–9. 10.18043/ncm.78.6.406 29203606

[16] Blue C Riggs S. Oral health care delivery within the accountable care organization. J Evid Based Dent Pract. (2016) 16 Suppl:52–8. 10.1016/j.jebdp.2016.01.016 27236996

[17] Koirala B Acharya S Spero L Mittal R Erchick DJ. A clinical competency framework for the basic package of oral care: perceptions of primary oral health providers in rural Nepal. Front Public Health. (2022) 10:914581. 10.3389/fpubh.2022.914581 35910900 PMC9330375

[18] Harnagea H Lamothe L Couturier Y Esfandiari S Voyer R Charbonneau A From theoretical concepts to policies and applied programmes: the landscape of integration of oral health in primary care. BMC Oral Health. (2018) 18(1):23. 10.1186/s12903-018-0484-8 29448934 PMC5815219

[19] Prasad M Manjunath C Murthy AK Sampath A Jaiswal S Mohapatra A. Integration of oral health into primary health care: a systematic review. J Family Med Prim Care. (2019) 8(6):1838–45. 10.4103/jfmpc.jfmpc_286_19 31334142 PMC6618181

[20] Gordon NP Mosen DM Banegas MP. Oral health care: a missing pillar of total health care? Perm J. (2021) 25. 10.7812/TPP/21.080 35348106 PMC8784083

